# 

**Published:** 1899-02-04

**Authors:** 


**Notices of Births, Deaths, and Marriages** are inserted in this column at a charge of 2s. 6d. for an announcement not exceeding four lines.

### NOTICE TO CORRESPONDENTS.

All MS., Letters, Books for Review, and other matters intended for the Editor should be addressed The Editor, "The Hospital," The Hospital Building, 28 & 29, Southampton Street, Strand, W.C.

The Editor cannot undertake to return rejected MS., even when accompanied by stamped directed envelope.

**Notice to Advertisers and Subscribers.**

All Advertisements, Orders for Copies of The Hospital, and Business Communications should be addressed to **THE MANAGER (Not to the Editor),** The Hospital, The Hospital Building, 28 & 29, Southampton Street, Strand, London, W.C.

The Cost of Subscription, post free, is as follows:—Per week, 2½d.; per quarter, 2s. 8d.; per half-year, 5s. 3d.; per annum, 10s. 6d. Foreign:—Per annum, 15s. 2d., payable, in all cases, in advance.

**NOTICE.—Vol. XXIV. of "The Hospital" (April, 1898, to September, 1898), in handsome cloth binding, is now on sale at the Office of this Journal, price 7s. 6d. Cases for binding the Numbers contained in this Volume 1s. 6d. Index to Vol. XXIV., 2½d., post free.**

**The Monthly Part for February is now ready. Price 6d., by post 8d.**

### THE STUDENT OF MEDICINE.

### APPOINTMENTS.

A. J. ANDERSON, M.B., C.M.Edin., appointed Medical Officer to the Workhouse of the Staines Union. A. Back, L.R.C.P.Edin., M.R.C.S.Eng., appointed Medical Officer for the Acle District of the Blofield Union. G. H. Baird, M.D.Brux., L.S.A., appointed Medical Officer to the Cable Department and Postal District, Charlton, also to H.M.S. "Monarch." A. F. Blagg, M.D., L.R.C.P., appointed Physician to the Orthopædic Hospital, Bristol. P. L. Booth, M.R.C.S., appointed Honorary Surgeon to North Lonsdale Hospital, Barrow-in-Furness. N. T. Brewis, M.B., F.R.C.P.Edin., appointed Gynæcologist to Leith Hospital. L. H. Bryson, M.B., C.M., M.R.C.S., L.R.C.P., appointed Medical Officer to the Derby School Board. J. Butler, M.B.-Glasg., appointed Junior Assistant Medical Officer to the Govan District Asylum, Hawkhead, Paisley. T. Carwardine, M.S.-Lond., F.R.C.S., appointed Surgeon to the Orthopædic Hospital, Bristol. E. Coleman, M.B., B.S.Lond., appointed Assistant Medical Officer of the County Asylum, Rainhill, near Liverpool. W. S. Dibbs, M.R.C.S., L.R.C.P.Lond., appointed Medical Officer for the Farnham Royal District of the Eton Union. J. G. Emanuel, M.B., B.S., B.Sc.Lond., M.R.C.S.Eng., L.R.C.P.Lond., appointed Resident Medical Officer to the City of London Hospital for Diseases of the Chest, Victoria Park. H. M. Eyres, M.B., C.M.Edin., appointed Medical Officer for the Scorton and Catterick Districts of the Richmond (Yorks) Union. G. W. S. Farmer, M.A., M.B., M.Ch.Oxon., F.R.C.S., appointed Honorary Surgeon to the Ratcliffe Infirmary, Oxford. J. H. Ferguson, M.D., F.R.C.P.E., appointed Ordinary Physician for Diseases of Women and Gynæcological Surgeon to Leith Hospital. F. R. S. Gaman, M.R.C.S., L.R.C.P.Lond., appointed Medical Officer for the First District and Workhouse of the Caistor Union. D. B. Hart, M.D., F.R.C.P.E., appointed Consulting Physician for Diseases of Women and Consulting Gynæcologist to the Leith Hospital. G. Kendrick, M.R.C.S., L.R.C.P.Lond., appointed Medical Officer for the Bilston No. 5 District of the Wolverhampton Union. C. R. Killick, M.B.Lond., appointed Medical Officer of the Workhouse and the Williton District of the Williton Union.

